# The paradox does not fit all: Racial disparities in asthma among Mexican Americans in the U.S.

**DOI:** 10.1371/journal.pone.0242855

**Published:** 2020-11-30

**Authors:** Guadalupe Marquez-Velarde

**Affiliations:** Department of Sociology, Social Work and Anthropology, Utah State University, Logan, UT, United States of America; University of Arizona, UNITED STATES

## Abstract

Mexican Americans have a lower prevalence of asthma than White Americans, Black Americans, and Other Hispanics. This is concordant with the Hispanic Paradox, which posits that Hispanics have good health and lower mortality than White Americans despite their relative socioeconomic disadvantages. However, the research is limited in relation to the effects of race on health, independent of ethnicity, among this population. In this study, the author disaggregated Mexican Americans, foreign-born and U.S.-born into two categories, White and Black Mexicans, in order to assess their likelihood of having an asthma diagnosis, compared to White Americans and to each other. This study used harmonized data from the National Health Interview Survey from 2000–2018 with a final analytic sample of N = 1,094,516. The analysis was conducted using binary logistic regression, controlling for acculturation and health behavior-related variables, as well as sociodemographic characteristics. In the results, Black Mexicans had a significant disadvantage in relation to their White counterparts and White Americans. The findings suggest there is an intra-ethnic racial disparity in asthma and the Hispanic paradox is not applicable across racial lines for Mexican Americans. These findings also suggest Black Mexicans’ poor asthma outcomes are the byproduct of various mechanisms of racial inequality.

## Introduction

According to the latest data from 2017 and 2018, asthma has a current prevalence of 7.9% and a lifetime prevalence of 13% in the United States [1, 2]. There are substantial differences by race and ethnicity. The current asthma prevalence for Hispanics is 6.4%, which is lower than the population prevalence and lower than the prevalence for White Americans (8.1%), Black Americans (10.1%), and Other non-Hispanics (6.7%). Among Hispanics, Mexican Americans have the lowest prevalence (5.1%) [2]. Thus, among Hispanics—and more saliently among Mexican Americans—we can observe the Hispanic Paradox in full effect. The Hispanic Paradox is the counterintuitive finding that Hispanics have better or comparable health and mortality profiles relative to non-Hispanic Whites (hereinafter called White Americans) despite their significant socioeconomic disadvantages [3, 4]. Within this literature, nativity is a strong predictor of positive health outcomes. For instance, Mexican immigrants have lower asthma prevalence than White Americans [5]. Foreign-born children and children of foreign-born mothers, as well as foreign-born Hispanics who live in neighborhoods with high concentrations of foreign-born residents, also have a significantly lower asthma prevalence [6, 7]. Although the asthma literature recognizes heterogeneity within Hispanic subgroups, Hispanics and in the case of this study, Mexican Americans, can be of any race. Yet, their experiences, including their health outcomes, have mostly been analyzed assuming racial homogeneity.

Race matters for health outcomes because it is associated with almost all diseases and mortality [8]. Fundamental cause theory suggests that individuals with higher socioeconomic status are able to use flexible resources, including money, knowledge, power, and prestige, in order to avoid illness and death [9]. In the United States, racial inequality largely determines socioeconomic status. The White-Black gap in median income, wealth, and college education has remained the same or has grown in the second half of the 20^th^ and early 21^st^ century [10–12]. These disparities in socioeconomic status (SES) in turn influence health status [8]. Furthermore, race has effects on health independent of socioeconomic status, and racial health disparities persist for most outcomes even after accounting for socioeconomic measures [13]. Racial discrimination is a stressor associated with many physical and mental health outcomes and health behaviors [14–16]. The physiological response to the cumulative stress of living in a highly racialized society leads to early deterioration, or weathering [17, 18]. Moreover, race also determines the quality of care patients receive. For instance, Black Americans receive poorer care than White Americans, regardless of health condition and clinical setting [19, 20].

Through racialization, groups are ascribed an arbitrary identity based on ideology, power, and stratification [21]. Hispanics, including Mexican Americans, are racialized primarily based on skin tone and socioeconomic status [22, 23]. Thus, assuming that all Hispanics, or even a subgroup such as Mexican Americans, have similar experiences and outcomes ignores racial inequalities within ethnic groups.

The literature on the health of Black or dark-skin Hispanics is somewhat limited. Yet, there is some evidence to suggest that they are disadvantaged in some health outcomes, including higher rates of diabetes [24] and higher prevalence of poor or fair self-rated health [25]. They are also less likely to have a usual source of health care [26]. Among Hispanic subgroups, Black Mexicans are more likely to report poor or fair self-rated health [27], and dark skin Puerto Rican men have been found to have higher systolic blood pressure [28]. Among Puerto Ricans, a study on gene-environment interaction found that among Puerto Ricans with high socioeconomic status, those with African ancestry have an increased risk of asthma in comparison to high SES Puerto Ricans of European ancestry [29].

This study aimed to expand on the body of literature of Black Hispanic health. The main questions answered in this study are:

1. Is there an even distribution of asthma at the intersection of race and ethnicity among Mexican Americans?
2. Are all Mexican Americans across racial lines benefitting from the health advantage postulated in the Hispanic Paradox literature?

## Methods

### Participants

Participants were men and women age 18 and over who participated in the National Health Interview Survey (NHIS) from 2000–2018. This cross-sectional household survey was first implemented in the United States in 1957 by the National Center for Health Statistics, a part of the Center for Disease Control. The main source of population health data in the US, the NHIS monitors trends and progress on a wide range of subjects, including illness, disability, health care access, and health behaviors [30]. Due to its wealth of demographic and socioeconomic indicators, the NHIS is suitable to study health disparities. The NHIS uses a multistage area probability sample design, in which clusters of addresses are selected from each state and the District of Columbia [30]. The survey is administered to 100,000 individuals in 45,000 households every year [31]. Updated every ten to fifteen years since 1957, the survey underwent a significant revision in 1996 and the sampling plan changes after the decennial census [30].

In order to use multiple data waves, from 2000 to 2018, I used the Integrated Public Use Microdata Series (IPUMS) NHIS, which uses the NHIS public use files in order to recode and harmonize the data over time. Since the survey goes through revisions, the variables in IPUMS NHIS have been integrated and made comparable across data waves. This process employs translation tables to reconcile coding schemes across years and integrates data from various NHIS public use files. IPUMS NHIS allows researchers to use data from multiple years without needing to concatenate data files. Furthermore, it provides consistent variable names across all survey years [31, 32]. I restricted the analytic sample to those who self-identify in one of the categories of the main independent variable. This yielded a sample of 1,094,516 respondents.

### Measures

The main independent variable in this study was racial and ethnic self-identification, a categorical variable coded 1 for White Americans (N = 777,294); 2 for Black Americans (N = 168,054); 3 for White Mexicans (N = 146,997); and 4 for Black Mexicans (N = 2,171). White and Black Mexicans self-identified ethnically as Hispanic of Mexican origin, and racially as White or Black. Race as measurement and lived experience is multidimensional. It is socially constructed through the interplay of self-identification, self-perception, other’s perception, physical characteristics, ancestry, among other processes [33]. Roth [33] argues that racial self-identification, what we select in official forms or surveys, is appropriate to study health outcomes because alike racial identity, they are both self-identification measures and as such, both are related to individuals’ experiences of race. Racial self-identification takes the form of a closed-ended survey question and that makes it a good measure to include in quantitative studies. Self-identification is particularly appropriate when the way individuals identify fits with official categorizations—in this case, those who strictly identify as White or Black [33].

Covariates of this study included acculturation measures, demographic and socioeconomic indicators, and health behaviors. Measuring the effects of acculturation is a prevailing practice in the study of Hispanic health, and in general, acculturation has a negative effect in health outcomes [34]. In order to assess the extent to which this is true in this study, I used two proxy measures of acculturation: nativity and length of residency, and language. The categorical variable of nativity and length of residency included native-born (1), foreign-born with less than five years of residency in the U.S. (2), foreign-born with five to fourteen years of residency in the U.S. (3), and foreign-born with fifteen or more years of residency in the U.S. (4). Language of interview included English (1), Spanish (2), or Bilingual in English and Spanish (3). I controlled for three demographic measures: age (categorical), gender (binary), and marital status (binary). Because higher socioeconomic status is usually associated with better health outcomes, it was also important to control for this variable [35]. The socioeconomic covariates were level of education (categorical), household income (categorical), and employment (binary). Finally, the last two independent variables were categorical measures of health behaviors: smoking and drinking, as these two health behaviors have been associated with the risk of asthma development [36].

**Fig 1:**
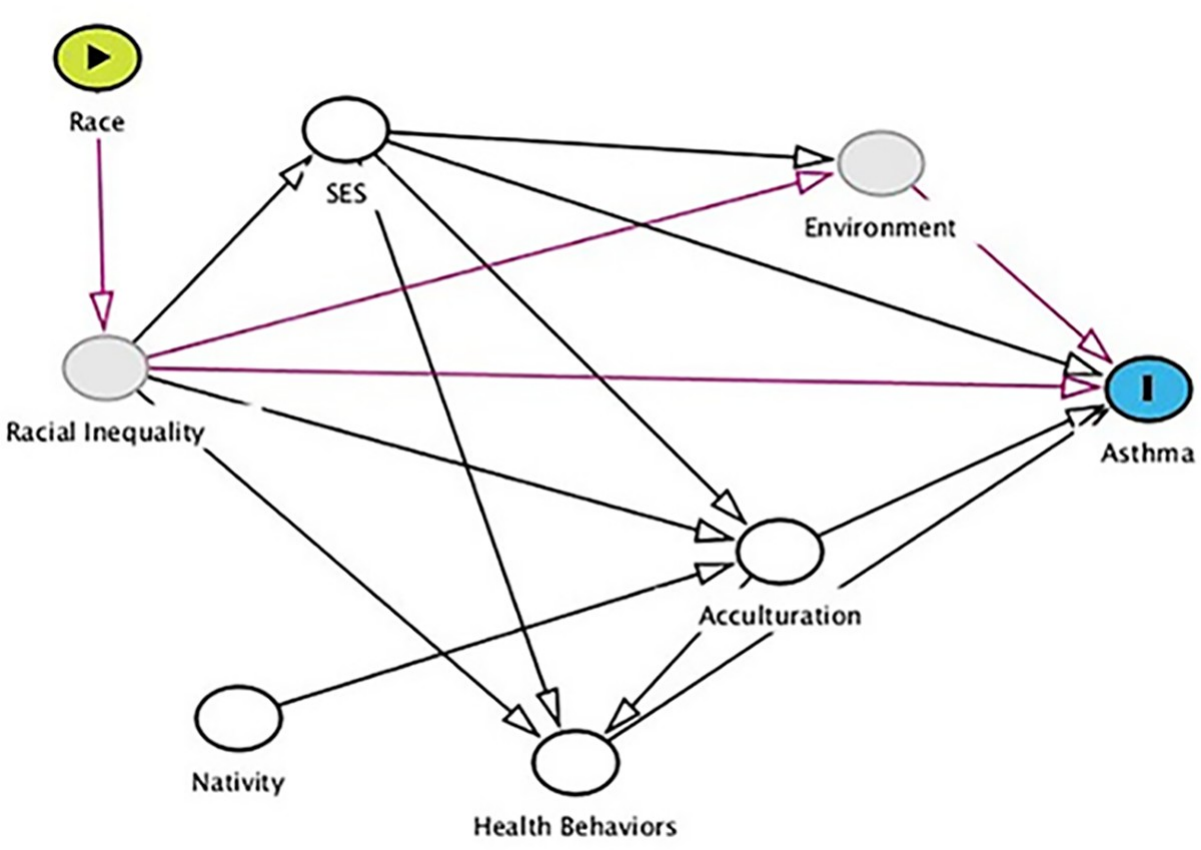


Finally, the outcome variable was a dichotomous measure of asthma (0-no; 1-yes). This measure identified adults in the sample who have been diagnosed with asthma by a doctor or other health professional. In the NHIS, asthma is defined as “a chronic respiratory disorder characterized by labored breathing and wheezing resulting from obstructed and constricted air passages” [32]. This variable has been included in the NHIS every year since 1997 for both the adult and children sample, and although the survey question has changed slightly over time, the wording has remained consistent since 1997: “Have you ever been told by a doctor or other health professional that you had asthma?” [32]. This measure is limited as it requires a proper medical diagnosis and those without access to healthcare might be undiagnosed, resulting in an undercount. In Fig 1, the directed acyclic graph (DAG) shows the association between the outcome, asthma, and the main independent variable, race. Race operates through racial inequality (unobserved) to influence covariates including socioeconomic status, health behaviors, immigrant acculturation, and environmental issues such as environmental racism (unobserved). In turn, all of these factors have an effect on asthma. Racial inequality and socioeconomic status are deeply intertwined; thus, SES also influences other covariates [37]. I created the DAG model using the online version of the DAGitty R package developed by Textor et al. [38].

### Statistical analyses

I used binary logistic regression to assess the effect of the independent variables on the probability of being diagnosed with asthma. Then, I performed a specification test by estimating a second equation after the full models. I used the right-hand side estimations from the full fitted models as predictors. In both cases, the z-statistic was significant, which indicates that the original models fitted adequately [39]. I used odd ratios, the exponentiated values of the logit coefficients, to interpret the effects of the predictors more intuitively [40]. Thus, results are discussed in terms of percentage and factor change [41]. I performed the analysis using Stata 15 [42] and employed the survey suite of commands (svy) in Stata to account for sampling weights, clustering, and stratification in the NHIS sample design [43]. Table 1 outlines the sample characteristics and Tables 2 and 3 display the multivariable analyses. In the first set of multivariable models (Table 2), I include in the analysis White Americans, Black Americans, White Mexicans and Black Mexicans, using White Americans as the reference group. In the models presented in Table 3, I only include both Mexican groups with White Mexicans being the reference category.

**Table 1:** Percentage distributions of sample respondents by racial/ethnic categories across all variables of interest.

	*NH White*	*NH Black*	*White Mexican*	*Black Mexican*
Respondents with Asthma	12.2%	13.2%	7.5%	13.2%
*Nativity and Length of Residency in the U*.*S*.				
US-born	95.1%	88.7%	45.7%	55.8%
FB less than 5 Years	0.5%	1.5%	5.2%	2.9%
FB 5–14 Years	1.0%	3.5%	17.1%	14.1%
FB over 15 Years	3.4%	6.2%	32.0%	27.2%
*Language*				
English	99.9%	99.9%	59.1%	70.2%
Spanish	0%	0%	25.0%	19.3%
Bilingual	0%	0%	15.9%	10.5%
*Age categories*				
18–34	26.9%	35.3%	45.4%	55.3%
35–49	26.7%	28.7%	31.1%	26.4%
50–64	26.0%	23.0%	15.8%	14.9%
65–80	15.8%	10.6%	6.6%	3.4%
80–85	4.6%	2.4%	1.1%	0%%
Female	51.6%	55.2%	48.5%	55.5%
Married	64.9%	42.1%	64.0%	58.9%
*Education categories*				
Less than HS	10.2%	18.2%	42.8%	33.7%
HS Grad	27.8%	30.7%	26.4%	29.2%
Some College	30.5%	32.7%	21.2%	29.3%
Bachelor's and higher	30.5%	18.5%	8.6%	7.9%
*Income categories*				
24,999 and below	19.7%	36.4%	31.2%	31.4%
25,000–34,999	10.1%	12.7%	14.4%	13.2%
35,000–44,999	9.5%	10.6%	12.4%	13.6%
45,000–54,999	9.0%	8.6%	9.9%	6.9%
55,000–64,900	7.5%	6.6%	7.2%	6.5%
65,000–74,900	6.7%	5.3%	5.4%	7.5%
75,000–99,999	19.6%	11.2%	10.9%	13.5%
100,000 and over	17.9%	8.6%	8.6%	7.4%
Employed	62.8%	60.3%	65.7%	68.2%
*Smoking status*				
Never smoker	54.2%	66.7%	73.5%	77.9%
Current every day smoker	16.8%	14.4%	7.1%	7.1%
Current some day smoker	3.6%	5.0%	5.8%	5.2%
Former smoker	25.4%	13.9%	13.6%	9.8%
*Drinking Status*				
Never drinker	16.6%	31.8%	31.8%	25.6%
Current drinker	68.5%	52.7%	56%	62.6%
Former drinker	14.9%	15.5%	12.2%	11.8%

**Table 2:** Logistic models of asthma controlling for race/ethnicity, acculturation, demographic, socioeconomic, and health behavior measures.

	*Race*	*Acculturation*	*Demographic*	*SES*	*Health Behaviors*
	*Odd Ratio (CI)*	*Odd Ratio (CI)*	*Odd Ratio (CI)*	*Odd Ratio (CI)*	*Odd Ratio (CI)*
*Ref*. *NH Whites*					
NH Blacks	1.094**	1.141**	1.045**	1.005	1.034*
	(1.061–1.129)	(1.106–1.177)	(1.013–1.078)	(0.973–1.038)	(1.000–1.068)
White Mexicans	0.588**	0.940*	0.865**	0.849**	0.876**
	(0.559–0.619)	(0.886–0.997)	(0.816–0.917)	(0.801–0.901)	(0.825–0.930)
Black Mex	1.097	1.543**	1.339	1.317	1.368
	(0.816–1.475)	(1.127–2.113)	(0.976–1.836)	(0.958–1.809)	(0.994–1.884)
*Ref*. *US-born*					
FB less than 5 Years		0.371**	0.350**	0.330**	0.341**
		(0.308–0.446)	(0.291–0.420)	(0.274–0.398)	(0.283–0.411)
FB 5–14 Years		0.392**	0.380**	0.376**	0.389**
		(0.349–0.439)	(0.338–0.427)	(0.334–0.423)	(0.345–0.438)
FB over 15 Years		0.543**	0.582**	0.581**	0.590**
		(0.509–0.580)	(0.546–0.621)	(0.545–0.620)	(0.553–0.630)
*Ref*. *English*					
Spanish		0.551**	0.565**	0.521**	0.535**
		(0.476–0.638)	(0.488–0.654)	(0.449–0.604)	(0.461–0.621)
Bilingual		0.666**	0.670**	0.613**	0.622**
		(0.568–0.780)	(0.571–0.785)	(0.525–0.716)	(0.532–0.727)
*Ref*. *18–34*					
35–49			0.811**	0.836**	0.803**
			(0.786–0.837)	(0.809–0.863)	(0.778–0.829)
50–64			0.822**	0.805**	0.758**
			(0.797–0.848)	(0.780–0.831)	(0.734–0.782)
65–80			0.714**	0.602**	0.561**
			(0.690–0.738)	(0.580–0.625)	(0.540–0.584)
80–85			0.485**	0.391**	0.369**
			(0.458–0.513)	(0.368–0.415)	(0.347–0.392)
Female			1.366**	1.318**	1.349**
			(1.334–1.399)	(1.287–1.350)	(1.316–1.383)
Married			0.839**	0.896**	0.884**
			(0.821–0.858)	(0.875–0.918)	(0.862–0.905)
*Ref*. *Less than HS*					
HS Grad				0.848**	0.856**
				(0.817–0.881)	(0.823–0.890)
Some College				1.045*	1.054**
				(1.007–1.084)	(1.015–1.094)
Bachelor's and higher				0.970	0.997
				(0.931–1.010)	(0.956–1.039)
*Ref*. *24*,*999 and below*					
25,000–34,999				0.896**	0.894**
				(0.863–0.930)	(0.861–0.928)
35,000–44,999				0.852**	0.853**
				(0.819–0.887)	(0.820–0.888)
45,000–54,999				0.833**	0.833**
				(0.797–0.871)	(0.797–0.871)
55,000–64,900				0.827**	0.828**
				(0.785–0.870)	(0.785–0.872)
65,000–74,900				0.829**	0.830**
				(0.784–0.876)	(0.785–0.878)
75,000–99,999				0.767**	0.776**
				(0.736–0.800)	(0.744–0.808)
100,000 and over				0.907**	0.926**
				(0.868–0.946)	(0.887–0.967)
Employed				0.747**	0.750**
				(0.726–0.768)	(0.729–0.771)
*Ref*. *Never smoker*					
Current every day smoker					1.123**
					(1.088–1.160)
Current some day smoker					1.057*
					(1.002–1.116)
Former smoker					1.236**
					(1.200–1.273)
*Ref*. *Never drinker*					
Current drinker					1.099**
					(1.062–1.138)
Former drinker					1.248**
					(1.200–1.299)

Notes: Confidence interval in parentheses.

** p<0.01

* p<0.05.

**Table 3:** Logistic models of asthma comparing White and Black Mexicans.

	*Race*	*Acculturation*	*Demographic*	*SES*	*Health Behaviors*
	*Odd Ratios (CI)*	*Odd Ratios (CI)*	*Odd Ratios (CI)*	*Odd Ratios (CI)*	*Odd Ratios (CI)*
*Ref*. *White Mexicans*					
Black Mex	1.865**	1.625**	1.547**	1.545**	1.563**
	(1.387–2.509)	(1.185–2.228)	(1.125–2.127)	(1.124–2.123)	(1.135–2.152)
*Ref*. *US-born*					
FB less than 5 Years		0.185**	0.191**	0.199**	0.202**
		(0.138–0.250)	(0.141–0.258)	(0.146–0.270)	(0.147–0.276)
FB 5–14 Years		0.274**	0.287**	0.299**	0.301**
		(0.225–0.334)	(0.234–0.352)	(0.241–0.370)	(0.243–0.374)
FB over 15 Years		0.442**	0.485**	0.500**	0.506**
		(0.390–0.499)	(0.429–0.548)	(0.442–0.567)	(0.447–0.573)
*Ref*. *English*					
Spanish		0.658**	0.650**	0.652**	0.673**
		(0.558–0.775)	(0.553–0.766)	(0.552–0.771)	(0.570–0.795)
Bilingual		0.723**	0.711**	0.687**	0.709**
		(0.613–0.853)	(0.602–0.839)	(0.582–0.812)	(0.598–0.842)
*Ref*. *18–34*					
35–49			0.892	0.909	0.875*
			(0.793–1.004)	(0.807–1.025)	(0.778–0.985)
50–64			0.952	0.921	0.856*
			(0.827–1.097)	(0.804–1.056)	(0.745–0.984)
65–80			0.775**	0.702**	0.627**
			(0.661–0.910)	(0.597–0.824)	(0.530–0.742)
80–85			0.772	0.701	0.639*
			(0.542–1.101)	(0.488–1.006)	(0.445–0.918)
Female			1.395**	1.318**	1.380**
			(1.269–1.533)	(1.197–1.453)	(1.247–1.527)
Married			0.786**	0.812**	0.795**
			(0.713–0.866)	(0.734–0.898)	(0.718–0.880)
*Ref*. *Less than HS*					
HS Grad				0.993	1.005
				(0.866–1.139)	(0.874–1.155)
Some College				1.277**	1.287**
				(1.124–1.450)	(1.132–1.464)
Bachelor's and higher				1.151	1.169
				(0.963–1.376)	(0.977–1.399)
*Ref*. *24*,*999 and below*					
25,000–34,999				0.947	0.939
				(0.822–1.091)	(0.814–1.084)
35,000–44,999				0.790**	0.795**
				(0.676–0.924)	(0.680–0.930)
45,000–54,999				0.850	0.857
				(0.709–1.019)	(0.714–1.028)
55,000–64,900				0.855	0.855
				(0.685–1.067)	(0.682–1.073)
65,000–74,900				0.966	0.977
				(0.792–1.178)	(0.799–1.195)
75,000–99,999				0.849	0.864
				(0.704–1.024)	(0.714–1.045)
100,000 and over				0.987	0.992
				(0.803–1.214)	(0.806–1.221)
Employed				0.785**	0.796**
				(0.709–0.870)	(0.716–0.885)
*Ref*. *Never smoker*					
Current every day smoker					1.119
					(0.953–1.314)
Current some day smoker					0.937
					(0.759–1.156)
Former smoker					1.398**
					(1.221–1.599)
*Ref*. *Never drinker*					
Current drinker					1.054
					(0.925–1.200)
Former drinker					1.159
					(0.996–1.348)

*Notes*: Confidence interval in parentheses.

** p<0.01

* p<0.05.

## Results

### Sample characteristics

In the descriptive analysis shown in Table 1, both Black Americans and Black Mexicans had the highest and equal rates of asthma (13.2%)—only slightly higher than White Americans (12.2%) but considerably higher than White Mexicans (7.5%). The vast majority of White Americans and Black Americans were born in the United States. Among Mexicans, 45.7% of White Mexicans and 55.8% of Black Mexicans were born in the U.S. Among the foreign-born, those who have resided in the U.S. for 15 years or more comprised the largest group. About 40% of White Mexicans and almost 30% of Black Mexicans were surveyed in Spanish or employed both English and Spanish. About half of White and Black Mexicans were in the 18–34 age group while White and Black Americans had higher proportions of individuals in older age categories. In terms of gender, the distribution was almost even. Black Americans and Black Mexicans had 55% of females. Black Americans had the lowest marriage rate (42.1%). White Americans had the highest level of education; about 30% had a bachelor’s degree or more. Only 18% of Black Americans and about 8% of Mexicans (both White and Black) had a college degree. Furthermore, 43% of White Mexicans had less than high school. Black Mexicans had higher rates of high school completion and some college (29.2–29.3%) than White Mexicans (26.4–21.2%). In terms of income distribution, Black Americans, as well as White and Black Mexicans, were mostly concentrated in the lower income categories while White Americans had higher proportions of people in the four highest income brackets. Over 60% of the sample were employed at the time of the survey, with the highest employment rate among Black Mexicans (68.2%). White Americans had the highest rate of current every day smokers and current drinkers, while about three quarters of White and Black Mexicans had never smoked. However, 56–63% were current drinkers, respectively.

### Multivariable models

#### Baseline models

In the first set of models considering race (Table 2), White Mexicans had a negative association with the outcome in relation to White Americans, the reference group. White Mexicans were 41.2% less likely to be diagnosed with asthma (0.559–0.619) whereas Black Mexicans were about 9% more likely to be diagnosed with asthma. Although the association was not significantly different than zero for Black Mexicans, it did show an opposite effect between White and Black Mexicans. Black Americans were more likely to be diagnosed with asthma and the association was significantly greater than zero (OR 1.094; 1.061–1.129).

#### Models with acculturation-related predictors

All foreign-born groups were less likely to have asthma relative to the U.S.-born and the associations were significantly greater than zero. This result is consistent with previous studies, which suggest that Mexican immigrants are less likely to have asthma [5]. The foreign-born group followed a gradient pattern in which the most recent immigrants were the least likely to have an asthma diagnosis (OR 0.37; 0.308–0.446) while those who have resided in the US for 15 years or more had the smallest advantage (OR 0.54; 0.509–0.580) vis-à-vis the U.S.-born; the same pattern continued once controlling for all covariates. This finding echoed the literature that has established a diminishing health advantage as time in the U.S. increases [34]. Those who were interviewed in Spanish or bilingually were less likely to have asthma and the associations were significantly greater than zero. Spanish speakers were 45% less likely to be diagnosed with asthma relative to those interviewed in English (0.476–0.638).

#### Models with sociodemographic predictors

All age groups were less likely to be diagnosed with asthma compared to the reference group, 18–34, and the associations were significantly greater than zero. An asthma diagnosis became less likely as age categories increased. Women were 36% more likely to be diagnosed with asthma than men (1.334–1.399). Those who were married were about 16% less likely to have asthma in relation to non-married individuals (0.821–0.858). In terms of education, respondents with less than a high school diploma served as the reference group. High school graduates were about 15% less likely to be diagnosed with asthma (0.817–0.881) while those with some college were 5% more likely to have asthma in relation to those with less than a high school education (1.007–1.084). Every income category was less likely to be diagnosed with asthma relative to those who earned $24,999 and below and all associations were significantly greater than zero. Those who were employed were also less likely to have asthma (OR 0.75; 0.726–0.768). Hence, those of higher SES were less likely to have an asthma diagnosis [35].

#### Models with health behavior predictors

All smokers were more likely to be diagnosed with asthma compared to the never smokers and the associations were significantly greater than zero; this is particularly salient for former smokers who were 23% more likely to be diagnosed. Both current and former drinkers were more likely to be diagnosed with asthma in relation to the never drinkers and the associations were also significantly greater than zero. Former drinkers were almost 25% more likely to have asthma (1.200–1.299). It is worth noting that being a former smoker or drinker had a greater effect on the outcome than those who currently smoke and drink.

#### Full models

After all predictors were included, White Mexicans were still less likely to be diagnosed with asthma than White Americans (OR 0.87; 0.825–0.930) whereas both Black Americans (OR 1.03; 1.000–1.068) and Black Mexicans (OR 1.36) were more likely to be diagnosed. The finding for Black Mexicans was near significance (0.994–1.884). Black Mexicans did not display a health advantage while White Mexicans did. Furthermore, the effect for Black Mexicans was greater than the effect for Black Americans. Thus, previous findings in regards to the Mexican asthma advantage [5] do not apply homogeneously to the Mexican population in the U.S. across racial categories. Black Mexicans were the most disadvantaged group in the analysis.

In assessing if there was a racial disparity within Mexicans (Table 3), we observed that after accounting for all covariates, Black Mexicans were 56% more likely to be diagnosed with asthma (1.135–2.152) relative to White Mexicans and the association was significantly greater than zero. The effects of all other independent variables followed similar patterns as the ones described above. There were less significant effects in terms of education, income, and health behaviors compared to the models in Table 2. In summary, Black Mexicans were more likely to be diagnosed with asthma than both White Americans and White Mexicans.

In complementary analysis (not shown in tables), I assessed if foreign-born Black Mexicans had a lower risk for asthma than U.S.-born Black Mexicans. At baseline, U.S.-born Black Mexicans were 2.72 times more likely to be diagnosed with asthma (1.516–4.909). Once all covariates were included in the model, U.S.-born Black Mexicans were still 89% more likely to have an asthma diagnosis than foreign-born Black Mexicans but this association was not significantly different than zero. Lastly, in assessing if there were significant differences between Black Mexicans and Black Americans, I found Black Mexicans to be 21% less likely to be diagnosed with asthma than Black Americans (0.637–0.974) at baseline. Once I added all predictors to the model, Black Mexicans were 16.5% more likely to be diagnosed with asthma than Black Americans but this association was not significantly different than zero. However, showing a disadvantage in the full model reinforces the argument that the Hispanic Paradox does not operate across racial lines, in this case among Black Mexicans.

## Discussion

This study aimed to make two main arguments. First, I ascertained that Black Mexicans did not have an asthma advantage as White Mexicans did. This finding is suggestive of an intra-ethnic health disparity. Hispanics (in this case Mexicans and Mexican Americans) are not racially homogeneous, and therefore, the Hispanic Paradox framework [3, 4] might not fit all Mexicans and other Hispanics across racial lines. White Hispanics and White Mexicans are able to capitalize from the prevailing system of racial stratification, which puts a premium on whiteness [22, 23].

This is evidenced beyond our findings as well. Puerto Ricans, for example, a group more closely associated with Black Hispanics [44] have a disproportionate asthma burden compared to other Hispanics and White Americans [45, 46]. Furthermore, the study of racial health disparities among Hispanics often aggregates all subgroups, which does now allow for a nuanced analysis of the effects of racial inequality within ethnic groups. Black Hispanic health literature is scarce [24–26] and much more rare within Hispanic subgroups [27–29]. This study contributes to that body of literature along with the scholarship on the Hispanic Paradox.

Second, the Black Mexicans’ health disadvantage is indicative of the racial inequality experienced by those who are perceived as belonging to the “Collective Black” in the U.S. [22, 23]. Racial processes, independent of ethnicity, shaped their health outcomes even after accounting for socioeconomic measures, as it does with Black Americans [13]. Racial inequality yields poor health outcomes through several mechanisms including socioeconomic deprivation, which makes them increasingly susceptible to live in urban, low-income, and crowded households [47]. As a result, there is higher exposure to indoor allergens, such as cockroaches, a strong predictor of asthma morbidity. In fact, Black Americans are 4 times more likely to be sensitized to cockroach allergen compared to White Americans [48]. Black Americans also experience a disproportionate burden of environmental hazards, or environmental racism. Those hazards also increase their risk of asthma [49]. Lastly, there is evidence that stress, including adverse life events and discrimination, is associated with asthma [50–52]. Thus, the distinct manifestations of racial inequality are likely to have a compounding effect in this population to establish and perpetuate asthma disparities.

Future research should continue to assess the role of racialization processes in ethnic populations. Population health research also needs to become more intersectional. There is an increasing need of health research at the juncture of race, ethnicity, immigration, sexual orientation, and other social characteristics.

This study has four main limitations. First, even though I used data from 2000–2018, the NHIS survey design is cross-sectional and therefore causality cannot be established. Furthermore, the survey varies across years. The harmonization of data afforded by the IPUMS version could allow for cross-temporal analysis in future work [53]. However, I did not integrate a cross-temporal analysis due to very small numbers of Black Mexicans from the year 2000 to 2008 (less than 100 and as low as 8). Second, the sample of Black Mexicans is small in relation to the other comparison groups. However, the subsample is large enough for this type of analysis and the number of predictors. From the Black Mexican group, 50.6% are foreign-born (1,086). This is significant given the size of the Black Mexican population in Mexico, which is estimated to be roughly 1.5 million [54]. Third, the outcome variable—that is, asthma—is dependent on a formal diagnosis by a health professional. Issues of uninsurance or underinsurance might prevent individuals from formalizing a diagnosis. Lastly, race is multi-dimensional and self-identification is only one dimension. Although the measure is appropriate for the outcome [33], having more than one dimension would strengthen this study.

In summary, I analyzed one health outcome, asthma, among White Americans, Black Americans, White Mexicans, and Black Mexicans, taking into consideration acculturation, demographic, socioeconomic, and health behavior covariates. Results indicated White Mexicans have an asthma advantage in relation to White Americans and Black Americans. Black Mexicans, on the other hand, were the most disadvantaged of the groups in the analyses. Based on these findings, this study produced two major takeaways: race has health effects independent of ethnicity among this population, and therefore the Hispanic Paradox is not applicable across all racial categories for Mexican Americans in terms of asthma. Secondly, I argue that the disadvantage Black Mexicans face is attributed to the racial inequality Black Americans and those adjacent to Blackness [22, 23] experience in the U.S. Drawing on the literature on racial health disparities and the Hispanic Paradox, this study expands the small body of work on health disparities at the intersection of race and ethnicity while contributing to a more intersectional perspective to the study of population health.
